# Rainbows over the world’s public health: determinants of health models in the past, present, and future

**DOI:** 10.1177/14034948221113147

**Published:** 2022-09-08

**Authors:** Oliver J. Dyar, Bo J. A. Haglund, Cecilia Melder, Tracey Skillington, Margareta Kristenson, Anna Sarkadi

**Affiliations:** 1 Department of Public Health and Caring Sciences, Uppsala University, Uppsala, Sweden; 2 Department of Global Public Health, Karolinska Institutet, Stockholm, Sweden; 3 Existential Public Health and Psychology of Religion, University College Stockholm, Bromma, Sweden; 4 Department of Sociology and Criminology, University College Cork, Cork, Ireland; 5 Medical Faculty, Linköping University, Linköping, Sweden

**Keywords:** Social determinants of health, health status disparities, socioeconomic factors, inequality, health policy, health promotion, education, environment, socio-ecological model, biopsychosocial model

## Abstract

The need to visualise the complexity of the determinants of population health and their interactions inspired the development of the rainbow model. In this commentary we chronicle how variations of this model have emerged, including the initial models of Haglund and Svanström (1982), Dahlgren and Whitehead (1991), and the Östgöta model (2014), and we illustrate how these models have been influential in both public health and beyond. All these models have strong Nordic connections and are thus an important Nordic contribution to public health. Further, these models have underpinned and facilitated other examples of Nordic leadership in public health, including practical efforts to address health inequalities and design new health policy approaches.

Apart from documenting the emergence of rainbow models and their wide range of contemporary uses, we examine a range of criticisms levelled at these models – including limitations in methodological development and in scope. We propose the time is ripe for an updated generic determinants of health model, one that elucidates and preserves the core value in older models, while recognising the developments that have occurred over the past decades in our understanding of the determinants of health. We conclude with an example of a generic model that fulfills the general purposes of a determinants of health model while maintaining the necessary scope for further adjustments to be made in the future, as well as adjustments to location or context-specific purposes, in education, research, health promotion and beyond.

## Introduction

### The importance of models in public health

We work with complex conditions in public health [1] so it is not surprising that a wide range of models has emerged within the public health arena. These models serve many purposes, including deepening understanding through simplifying complexity and making the previously implicit explicit, facilitating communication, and allowing predictions and simulations [2]. The core models that emerge and persist within a discipline (e.g. Prochaska’s stages of change model, Kolb’s model of learning) [3, 4] are also a way of illustrating the central dynamics in prevailing theory, of illuminating uncertainties, and of discovering new questions. These core models therefore have important implications for how a discipline evolves over time.

Models visualising the determinants of health are among the most widespread and influential models in public health. Examples include the psychobiological model by Marmot and Wilkinson [5] and Diderichsen’s model [6] on how inequalities are created by individuals’ living conditions. Perhaps the most widely recognisable of these determinants of health models is the rainbow model [7]. Developed first by Haglund and Svanström in the 1980s and then reaching a wide audience after publication by Dahlgren and Whitehead in 1991, rainbow models have subsequently become a symbol for public health work in general, as Dahlgren and Whitehead recently reflected [8].

### Rainbow models: an important Nordic contribution to public health

Dahlgren and Whitehead’s rainbow model, as well as other rainbow models that came both before and after it, have strong Nordic connections and are thus an important Nordic contribution to public health. As well as playing a role in how the social determinants of health have become generally accepted as part of most theories in public health, these models have also underpinned and enabled other examples of Nordic leadership in public health – in particular practical efforts to address the social determinants of health [9].

The historical significance for the field of public health, and the ongoing relevance of these rainbow models inspired us to write a commentary article for this special edition of *SJPH* with the objective of reflecting on the emergence and evolution of these particular models; it struck us that for such influential and widely used models, surprisingly little has been written about their development. We begin our commentary by exploring the context for their emergence. Next, we review criticisms levelled at these models, both in terms of methodological development and scope, before discussing their endurability in a wide range of contexts – despite these criticisms. We conclude by suggesting that the time is ripe for developing an updated rainbow model, and we present a tentative starting point drawing on discussions among the authors that have occurred throughout the drafting of this commentary.

## The past: development of determinants of health models

### A growing awareness of the social determinants of health

The emergence of rainbow determinants of health models was closely linked to the growing popularity of the concepts of *community diagnosis* and *supportive environments for health* in the mid-late 20th century, concepts resulting from important trends in public health over the preceding centuries. Throughout the 17th and 18th centuries there was a growing scientific and political awareness that social factors had a role to play in determining the health of individuals and a population. For instance, the Swedish Collegium Medicum stated in 1755 that district medical officers should record annual patterns of epidemic and endemic diseases as well as morbidity and mortality. In addition, the officers were encouraged to describe the important factors which they believed influenced these disease patterns in terms of demography, living conditions, environmental health hazards, as well as behaviours [10]. In the 1840s several European countries conducted studies on the effects of equity public health reforms, which had been introduced to improve sanitation, housing, and worksite health promotion. Rudolf Virchow in Germany was one of these early champions in clarifying the effects of poverty and working conditions on population health [11], and Edwin Chadwick was a social reformer with similar influence in the UK on the social determinants of health, known for his work to reform the Poor Laws and to improve sanitation and public health [12].

Several developments occurred during the mid-late 20th century that laid the foundation for the emergence of rainbow models. The empirical evidence base for the role of social determinants of health strengthened through a range of scientific studies, for instance those of Thomas McKeown during the 1970s. McKeown concluded from his analyses that ‘in order of importance the major contributions to improvements in health in England and Wales were from limitation of family size [a behavioural change], increase in food supplies and a healthier physical environment [environmental influences] and specific preventive and therapeutic measures’ [13]. In a political context, the 1980s also saw the publication of the Black report in the UK, documenting inequalities in mortality rates for men and women between socioeconomic groups in different parts of the country, and between racial groups. The report also identified inequalities in access to health services, particularly preventative services, with low rates of uptake by the working classes [14]. The group’s recommendations focused on increased government intervention and spending in community health and primary care, as well as broader social policies such as increasing child benefit, improving housing, and agreeing on minimum working conditions with unions. In retrospect, these recommendations were beginning to highlight the role of supportive environments for health.

Over a similar time period in the mid-20th century, the community diagnosis concept was introduced in the epidemiological literature by Morris, as a tool for policy development in dealing with inequity in health [15]. Broadly, community diagnosis involves developing a quantitative and qualitative description of the health of a community and the factors which influence their health, identifying problems, proposing areas for improvement and stimulating action. Community diagnosis as a concept was first presented in the context of developing countries by King in 1966 in his book *Medical care in developing countries*. . . [16]. Dever introduced a model for developed countries in 1980 that shared similarities with the concept of community diagnosis, entitled *Holistic health – an epidemiological model for policy analysis* [17].

### Early rainbow models by Haglund and Svanström (1982–)

The Community Health Unit in Skövde, Skaraborg county, was at the forefront of public health activities in Sweden in the 1970s. This was a time when need and goal-oriented healthcare planning and health policy discussions were becoming more popular in Sweden, and were officially expressed by the Congress of the Federation of Swedish County Councils in 1979 [18]. The need for epidemiologically based planning was becoming the focus and the community diagnosis model the natural choice both for developing and developed societies [19]. As an illustration, one important part of the community diagnosis is identifying the stakeholders and their role in a process of change in health action programmes. In the community and health profiles of Skaraborg county two of the 17 municipalities deviated in indicators of mental health. These municipalities had many furniture factories where organic solvents (with known adverse effects on mental health) were used in painting the furniture. Experiences of practical safety work were at that time lacking [20, 21]. The change process included stakeholder activities such as education with unions for better safety procedures and environmental changes of the production line, such as reusing paint to make the production process both safer and cheaper.

**Figure 1.**
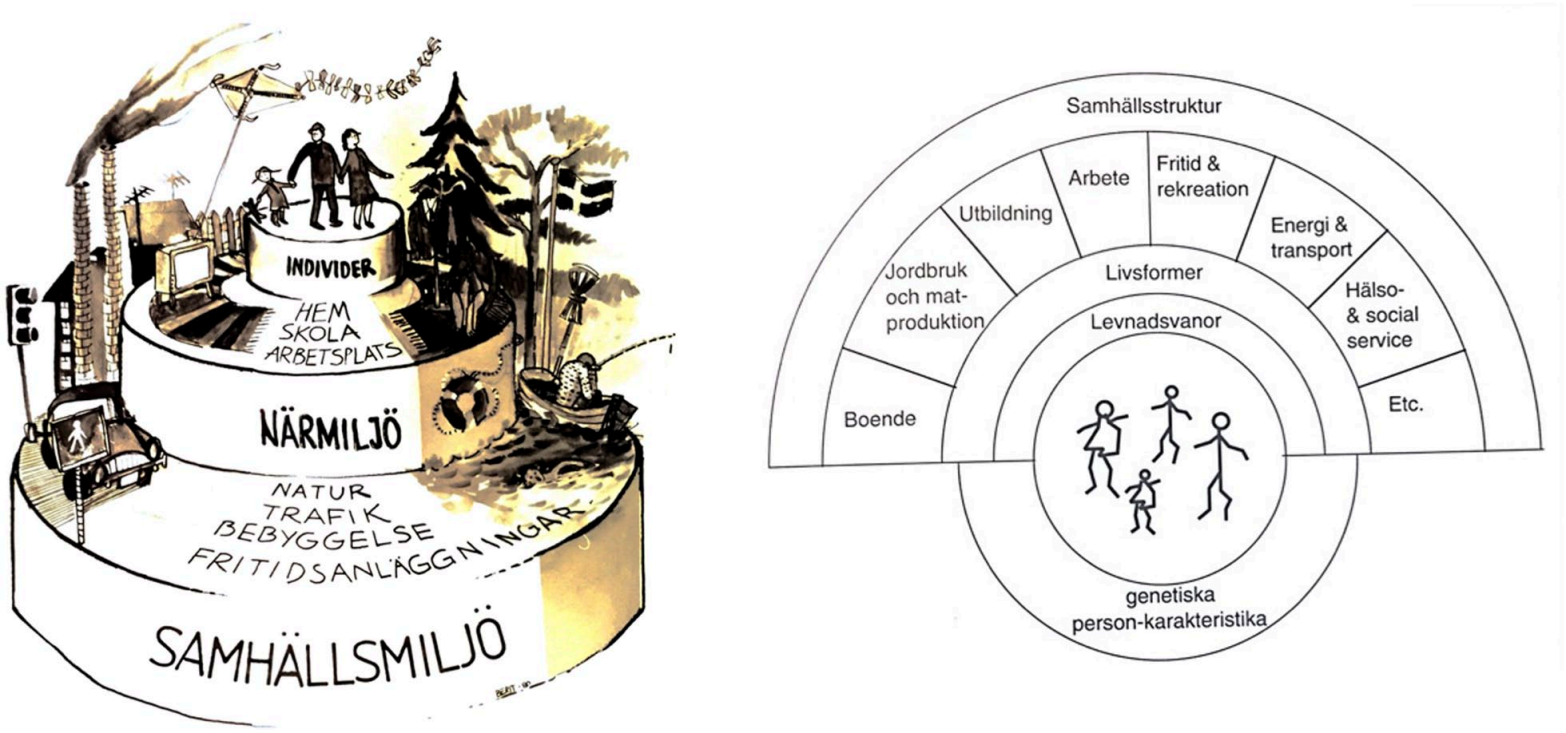
Early versions of determinants of health models. The cake model from the early 1970s (left) and the first rainbow model from the early 1980s (right) [23, 31].

In the context of these activities at the Community Health Unit, a number of models of determinants for health were discussed. The first model used in community intervention and policy discussions at the unit was the so-called ‘cake model’ for public health actions during the beginning of the 1970s (Figure 1, left). Leif Svanström and Bo Haglund, working at the unit in the 1970s, were both members of a Nordic network related to the medical student journal *Motpol*. At one of their meetings in Copenhagen, a first rainbow model was drawn in the sand in a garden (Figure 1, right), in order to better appreciate how to enhance understanding of the social determinants of health. The model evolved over the following years and was first presented in Swedish in 1982 in a report from a national seminar arranged by Sjukvårdens Planerings och Rationaliseringsinstitut (SPRI) [22] and then published in a textbook of community medicine in 1983 [23], as well as in English [10], and in Haglund’s doctoral thesis about community diagnosis in theory and praxis [24].

These early action-oriented rainbow models had theoretical inspirations both from Uri Bronfenbrenner’s first socio-ecological models [25] and the writings of the educator, philosopher and pedagogic thinker Paulo Freire who presented a model of *see, analyse and act* in his 1968 book, *Pedagogia del oprimido* [Pedagogy of the oppressed] [26]. In the context of the determinants of health models, ‘see’ became the community (self-)diagnosis; ‘analyse’ referred to the use of the model; and ‘act’ could be implemented with participation of those affected. Actors for prevention and health promotion could be occupational health or primary healthcare. The model could be used as a framework for different types of actions aiming for better health and wellbeing of the population, defining actors for disease prevention and health promotion in different settings [23]. That Haglund and Svanström were considering such themes at this time was closely related to the consequences of activism and lobbyism among medical students in Sweden over the previous decades, particularly under the banner of *Aktionsgruppen för fredstjänst* [Action group for peace service] [27]. These efforts had led to the establishment of Sandöskolan in 1971, a one-year educational programme aiming to prepare young Swedes for international work, as a publicly funded non-military alternative to compulsory service with the armed forces [28]. The training included a 6-week course in tropical medicine in Uppsala/Stockholm. The focus on third world countries had a major impact on the future lives of many students, of which Haglund was one, 1973–1974. It was in this context, for instance, that Haglund was introduced to King’s book *Medical care in developing countries. . .* [16]. Other future graduates included Hans Rosling, Kjell Asplund, Staffan Bergström, Jerker Hetta, Lars Klareskog, Lars-Åke Persson, Anders Wahlqvist and Claes-Göran Östensson.

Further iterations of the early rainbow model were developed during the 1980s, including in a 1986 textbook by Svanström and Haglund: *Att förebygga: samhällsmedicin i praktiken* [To prevent – community health in praxis] [29]. There, the perspective of prevention at the micro and macro levels were illustrated in 12 layers from the individual to the global level, based on a 1977 book by Edqvist [30]. These levels were subsequently reduced to six in a model presented for the development of strategies and evaluation of the Stockholm Cancer Prevention Program, on the basis that fewer layers enabled an easier understanding of the social determinants of health. The original sand-drawn model was included in the 1992 textbook *Public Health Science – an introduction* (in Swedish) [31], and a rainbow model was featured on the front page of the second version of the 1983 textbook, published in 1995. During this time Leif Svanström was head of the department of social medicine, Kronan, Karolinska Institutet and Bo Haglund was university lecturer at the department, as well as deputy chief medical officer in public health at the Stockholm County Council.

### Dahlgren and Whitehead (1991)

**Figure 2.**
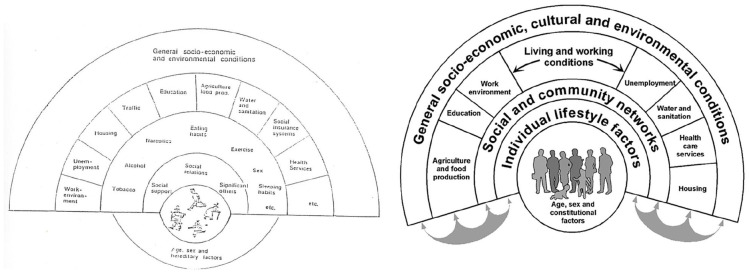
Dahlgren and Whitehead rainbow model 1991 (left) and 2007 (right) versions [7, 35].

In the late 1980s and early 1990s Margaret Whitehead was a doctoral student at the department of social medicine, Kronan, Karolinska Institutet. At the same time Göran Dahlgren worked with healthcare planning in the Stockholm County Council. In 1991 Whitehead and Dahlgren presented their rainbow model (Figure 2) as part of a discussion paper for the World Health Organization (WHO) regional Europe office [32], with many parallels to the earlier models of Haglund and Svanström. The model was created to suggest that quite distinct levels of intervention are needed for health policy making, and was eventually published by the Institute for Futures Studies, Sweden in 1991 [7]. The rest is history, so to say, as described in Dahlgren and Whitehead’s recent commentary [33], and the rainbow model has been recognised as one of the 50 key achievements in the past 50 years of social science research by the UK’s Economic and Social Research Council [34].

## Criticisms of rainbow determinants of health models

Many criticisms have been levelled at rainbow models over the years, in particular concerns around definitions, and limitations in terms of methodological development and scope.

### Definitions of determinants of health (or the lack thereof)

Early rainbow models do not appear to have been developed with an explicit definition of determinants of health. It appears that early rainbow models draw on the WHO’s definition of health as being ‘a state of complete physical, mental and social well-being and not merely the absence of disease or infirmity’ [36]. This means that self-rated measures of health and wellbeing are accounted for, not merely measures of morbidity and mortality. Some more recent suggestions for updating rainbow models [37–39] seem to move towards a broader vision of health determinants grounded in supportive environments and sustainable development goals (see the Sundsvall statement (1991) [40]), as well as calls for integrated action at the levels of individuals, communities and society as described in the Ottawa charter (1986) [41].

In their 1991 paper, Dahlgren and Whitehead described determinants as the main influences on health, stating that they can be loosely categorised into factors that threaten, promote or protect health [32]. The Commission on Social Determinants of Health framework describes *structural determinants* as those that generate or reinforce social stratification in society and that define individual socioeconomic position, and use that term, rather than *distal factors*, to highlight the causal hierarchy of social determinants involved in producing health inequities [42]. More recently Dahlgren and Whitehead suggest that there is a difference between *drivers* and *determinants*, using this division as a justification for not including proposed ‘commercial determinants’ or ‘racism’ in a determinants of health model [8]. However, they did not go on to provide a clear method for distinguishing between determinants and drivers.

### Limitations in methodological development

As the historical account above illustrates, the initial rainbow models (up to and including Dahlgren and Whitehead’s model) were the result of the focused work of a small number of individuals, drawing on their expert knowledge and opinions, primarily to portray the complexity of health determinants – but lacking a transparent, validated and reproducible methodological development process (for instance, a Delphi procedure).

Similarly, although rich epidemiological evidence supports the associations and predictive capacity between determinants of health and outcomes – independently as well as examined together – the early rainbow models were not developed to reflect every single such relationship, nor to demonstrate the potential lack thereof. The main reason for this was of course that the model primarily served educational, communicative, and policy influencing purposes. As a model in such widespread use, however, reasonable demands are continuously placed on its comprehensiveness and sensitivity to current debate and policy relevance, which has led to it being criticised for having an insufficiently explicit evidence base.

### Limitations in scope

An increasingly important limitation of these early rainbow models concerns their comprehensiveness. As a model of health, they represent an abstract and simplified view of reality; a map is never a direct copy of a territory, but it is vital that a map incorporates the most important aspects of a territory. In recent years several modifications have been proposed based on the contention that the existing models (typically referring to Dahlgren and Whitehead’s model) fall short, either through not including a potentially important type of determinant at all (e.g. digital technologies) [39] or through concerns about including a determinant implicitly by containing it within other related determinants (e.g. the wide ranging influence of relevant environmental determinants, or spiritual aspects) [37].

There have naturally been many developments in the world over the three decades since Dahlgren and Whitehead’s rainbow model was first presented [8, 42–44]. While it would not be feasible to summarise all these here, some illustrative examples can be provided. A particularly significant development has been the digital revolution and the social acceleration of the information age, with the widespread consequences this has had for the structure and functioning of society, culture, politics, education, entertainment, and the economy. Recognising this, Rice and Sara recently suggested adding a separate outer ring to Dahlgren and Whitehead’s model, ‘information and communication technologies’ [39], while Morley et al. proposed incorporating the ‘infosphere’ as a determinant of health through its inclusion in all four layers of the same model [45]. The COVID-19 pandemic has clearly illustrated the potential impacts on health of the infosphere and digital literacy, particularly in societies that have embraced the use of digital tools across many arenas of modern life with risks for digital and social exclusion, for instance in booking vaccinations and test appointments [46, 47].

Other changes over the past decades that have significantly impacted on the health of populations include the rapid expansion of displaced people and migration globally, as comprehensively evidenced in the 2018 Lancet Commission [48]. The number of international migrants increased from 84 million in 1970 to over 280 million in 2020, an increase from 2.3% to 3.6% of the world’s population [49]. Currently, a variety of cultures and ethnicities exist intertwined within countries, communities, families and even within one and the same person. This reality has relevance for all levels in the rainbow model, on the family tradition, eating habits, reliance on healthcare and even relation to life itself. Globalisation has also led to the emergence of transnational corporations which cast daily influences on individuals through what are increasingly referred to collectively as the commercial determinants of health [50, 51]. Similarly, there has been a growing awareness of the impact of governance on health, and what are often termed the political determinants of health [52, 53]. These factors are naturally interconnected, for instance as evidenced in debates on neo-colonialism.

A deeper understanding of the relevance of environmental factors has also emerged over the past three decades, during which we have begun to see the real-world impacts of climate change on health [54]. In the context of urban planning and the built environment, several issues have gained significant attention internationally and provoked important debates on the city as a supportive environment for health [54, 55], including the lack of green spaces in large urban areas, racially discriminatory housing policies, the overuse of heat-retaining building materials (such as concrete or asphalt), and the low availability of tree canopy and other cooling mechanisms. Changes occurring in the global (rising global temperatures) and local environment (pollution levels) intersect with those arising in the built, social and cultural environments (e.g. ritual practices of everyday life, development, community engagement, etc.). All, in turn, interact with the more individual determinants of health (age, lifestyle). Apart from events with the potential for sudden impacts on health (e.g. heat waves, floods, hurricanes, wildfires), climate change also contributes to the gradual loss of traditional ways of interacting with nature, community life, economic security and cultural heritage which, in turn, have an impact on migration rates [56].

Similarly, the existential health dimension, consisting of religious, spiritual, and secular meaning-making systems [57], has received increased attention in research and practice over the past decades [58, 59] after correlations between a large variety of health outcomes and the existential dimension were first established in epidemiological population studies primarily in the United States in the 1970s and 1980s [60, 61]. The WHO’s quality of life questionnaire [62] has been revised and extended, for instance, to include eight existential aspects covering spiritual, religious and personal beliefs. Melder has proposed extending the rainbow model to explicitly incorporate these existential aspects as significant determinants of health, especially in the context of increasingly broader definitions of health [63]. These aspects encompass a range of factors, including individual attitudes to hope, beliefs and trust related to health and life on the primary micro-level; supportive environments in the community on the secondary meso-level; and international organisations and government health policies on the tertiary macro-level [64, 65].

Beyond the comprehensiveness of determinants that are considered relevant for health, additional criticisms have been made on the (lack of) representation of the relationships between determinants, both within and between layers. However, the more comprehensive and specific the model, the more visually complex it will become, potentially compromising its utility and breadth of relevance – as experienced with an early model that contained 12 different rings.

## The enduring relevance of rainbow models in different contexts

Over the years other determinants of health models have emerged, including the psychobiological model by Marmot and Wilkinson [5] and Diderichsen’s model [6] on health inequalities. These important models address some of the criticisms of rainbow models discussed above (e.g. connection with an empirical evidence base), but these models also have narrower intended purposes and range of applications than rainbow models. Indeed, rainbow models appear to have an enduring relevance in many contexts now, in part reflecting the widespread acceptance of the impact of social determinants on the health of individuals and populations. Within policy and politics, the Dahlgren and Whitehead rainbow model has been extensively used in policy discussions around the world [8]. Rainbow models support efforts to implement a *health in all policies* approach by ensuring that responsibility for public health work is understood as a whole of government responsibility rather than for the health sector alone; this is particularly well illustrated in Norway with a new law (Public Health Act 2012), that spans across different levels of society aiming at a reduction of social health inequalities by adopting a health in all policies approach [66].

In education, it is not surprising that rainbow determinants of health models are now to be found in most public health textbooks, given their purpose is to visually represent the main determinants of health of populations [67]. The models are an effective pedagogical tool that capture several important aspects of public health theory and practice: that public health focusses on the causes of ‘health’ and not exclusively on the risk factors for individual diseases; that these determinants are in so many cases the same for the most common diseases; that many of these determinants are to be found outside the formal health services, and thus require collaborative multi-sectorial efforts; and that many of these ‘causes of the causes’ are social factors which can herd together and lead to the emergence of inequalities in health [42, 68]. Rainbow determinants of health models are also commonly encountered in public health research, despite the limitations in their own empirical evidence base discussed above. Examples include supporting the problem formulation stage (for instance, two recent doctoral theses from Sweden [69] and Norway [70]); as a logic model in systematic reviews to provide a structure on which to identify different study types and to present their findings [33, 71]; and even in semistructured interviews, for instance to explore individual attitudes to the prevention of illness, including agency and where responsibility lies [72]. These diverse uses illustrate the value of the model for helping expanding perspectives outwards and accounting for different layers of influence on health [8].

## Updating the rainbow model in a Swedish context: the Östgöta model (2014)

**Figure 3.**
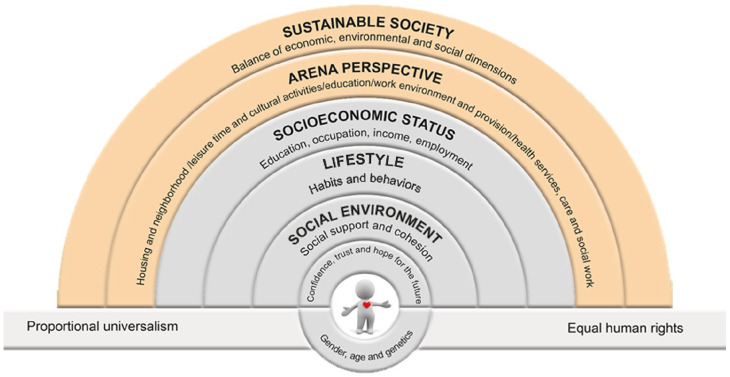
The Östgöta model for equity in health – an interplay between individual, environment and society. For the Östgötakommissionen. Kristenson and van Vliet based on the model by Dahlgren and Whitehead [38].

Many of the criticisms of rainbow models recounted above were addressed during the development process of what has come to be known in Sweden as Östgötamodellen, the Östgöta model. This model (Figure 3) was developed as part of the Östgöta Commission for Health Equity which worked from 2012 to 2014 in Östergötland county, Sweden [38]. The commission was launched by politicians in the county and in the 17 local municipalities who at the time worked together in a governance called ‘Östsam’ steered by a coalition representing all political parties, and was inspired by *Closing the gap in a generation*, the global WHO commission for health led by Michael Marmot [23]. The Östgöta Commission was composed of 10 researchers with a broad range of scientific backgrounds, two politicians and two civil servants, and was chaired by Margareta Kristenson. The work started with a community diagnosis, including an analysis of life conditions and socioeconomic differences in health in the county and in local municipalities (using empirical data for a broad range of health measures, including from self-rated health, dental caries, sick leave, myocardial infarction, and years of life lost). Inequalities in health in all measures were examined and identified for a range of measures of socioeconomic status in this ‘welfare’ county. Politicians from all parties were very eager to have a greater understanding of why this was the case and what could be done to improve the situation, and the commission and the politicians had the same clear aim for the subsequent work.

A series of workshops and dialogues with local politicians, community actors and inhabitants was organised over a period of 2 years. This included discussions of possible explanations of observed inequalities in health, grounded in international research and two established theoretical models: the psychobiological model by Marmot and Wilkinson [5] and Diderichsen’s model [6] on how inequalities are created by individuals’ living conditions, and in the former how this is translated and mediated by psychosocial and physiological mechanisms. These models helped define and understand both the more proximal and individual determinants of health, and also the distal, structural determinants, that is, the ‘causes of the causes’ of poor health outcomes [42]. However, these models were not well suited to the pedagogic needs at hand, being both too theoretical and insufficiently explicit. In contrast, the rainbow models proved more useful for illustrating the complexity of health determinants in a pedagogically coherent manner, and for providing a ‘map’ of the determinants.

During the Östgöta Commission it became apparent that there was a need to update the existing rainbow models. This was achieved through dynamic interactions between the commissionaires, politicians, and civil servants, led by the chair and the chief officer for public health, Jolanda van Vliet. From this reflection process, the Östgöta model (Figure 3) was born with the purpose of visualising the complexity of determinants that needed to be acted on and their relationship between one another, stressing the two-way interaction.

As equity in health is dependent on a sustainable society, this label was chosen for the outer layer of macroeconomic, social and democratic structures, with the subdomains representing economic, ecological and social dimensions. The label ‘arena perspective’ was chosen for the second outer layer, corresponding with the ring labelled ‘living and working conditions’ in Dahlgren and Whitehead’s model, emphasising a major strategy underlying the following policy recommendations, that is, applying an arena perspective, meaning that we ‘define, analyse and support the living conditions/arenas which people are born, grow, live, work and age and die, with the aim to give preconditions for equity in health’ [42]. These arenas include housing and neighbourhood, leisure time and culture, school and learning, work and income, and also the welfare system in terms of health and social services. Specific policy recommendations were then developed for each arena. Below this, a new layer was added which explicitly described individuals’ socioeconomic status, to illustrate and reinforce that these comprise several different domains (i.e. not simply education, but also income and occupation). While this essential domain in work to improve population health is dependent on the two outer societal layers, it is also a main determinant on the following inner layers of health determinants. Individual lifestyle factors, in terms of habits and behaviours, was placed directly below the socioeconomic status level, followed by a layer on the individual’s social environment, which was developed to include social support and the bonding, bridging and linking dimensions of social cohesion, as described by Green et al. [73].

Below this, two new dimensions were included to incorporate essential psychological and psychobiological factors, areas where research has considerably developed over the past decades; earlier models only included social factors and lifestyle. For instance, recognising the importance not only of psychosocial risk factors, but also of the independent protective effects of psychological resources against somatic disease and for health in a broader sense [74–76]. Indeed, the essential psychological resources (confidence, trust, and hope for the future) were defined by the politicians as the centre core for the work and were seen as prerequisites for equity in health. Finally, it was important also to illustrate the psychobiological mechanisms related to these effects and to social inequality, such as hypothalamic–pituitary–adrenal (HPA) axis dysfunction and chronic low-grade inflammation. Therefore, a new layer was added below this to incorporate essential psychological resources, (confidence, trust and hope for the future), and a red heart was inserted in the inner circle to symbolise the link to psychobiological mechanisms of vulnerability and resilience [74, 77, 78]. An important component of this model is the underlying understanding of the relationship between variables and layers. Beyond developing a model that was comprehensive, easy to understand, and explicit in its focus, it was also important to convey where the primary influence of policies lay. To do this, a colour code was added to illustrate how the main focus for politicians and policies was the outer two layers of society and societal arenas, coloured orange, with the more individual-oriented inner circles coloured grey.

Apart from its pedagogic value, the Östgöta model has also been extremely valuable in practical terms. For instance, in communicating with politicians and policy makers, not only during the work of the commission in Östergötland county, but also afterwards in policy planning. The model has since been included in the final report from the Swedish national commission on equity in health [79], and appears in the main Swedish textbook on social medicine and public health [67].

## A need for an updated rainbow model

We have chronicled the emergence of various rainbow models of determinants of health and illustrated how these models are now used in different settings. In line with the first half of George Box’s maxim that ‘all models are wrong, but some are useful’, it is not surprising that challenges have been levelled at existing models over the years, and that these challenges have occasionally crystalised in concrete suggestions for new models. We have also discussed throughout this commentary a variety of ways in which the second half of Box’s maxim also still holds true when considering the ongoing influence of rainbow models, despite the limitations of a particular model. Indeed, most of the suggested changes and updates that we have encountered can be regarded in many respects as adjustments made to the ‘core’ elements of the rainbow model. This strongly suggests that these rainbow models capture and convey certain enduring truths and can be seen as an important Nordic contribution to public health.

We suggest that the time is now ripe for a consideration of how these enduring truths speak to a range of newer health concerns arising as a consequence of various epidemiological, demographic and ecological transitions that have occurred across the world over the past 30 years. To begin to address these issues and, indeed, the challenges created by a lack of clear definitions, we suggest that the primary purpose of a revised model should be to clarify and communicate how a wide range of factors potentially influence the health of a population, and to illuminate the multiple entry points for interventions and policies designed to improve population health resilience (through addressing risk factors or utilising protective resources for physical, mental and social wellbeing, morbidity and mortality). An optimal generic model would efficiently fulfill this intended purpose while also allowing scope for further adjustments as the ‘determining capacities’ of different factors change over time, as well as facilitating location and context-specific adaptations of the model (e.g. in education, research, health promotion, or further policy development).

**Figure 4.**
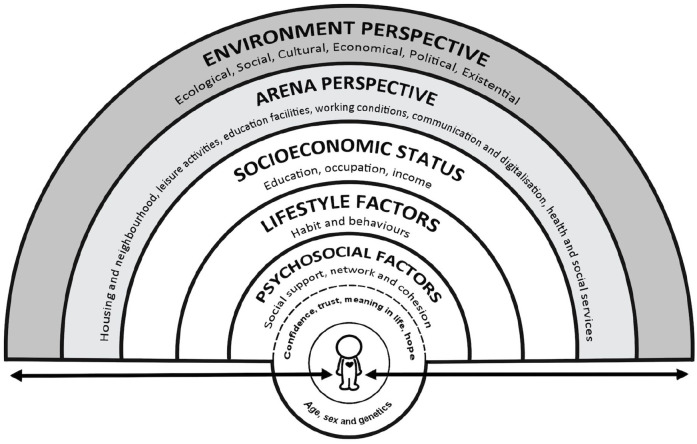
Generic determinants of health model.

Optimally, the development of an updated model would occur through a formal consensus procedure with the participation of experts from a range of relevant disciplines and would explicitly refer to the relevant empirical evidence base where possible. Further, there is a need to give more critical attention to visualising the dynamic relationship between determinants; rainbow models have a tendency to suggest the relationship between variables is fixed, thereby failing to capture the dynamic element of their interaction which, in turn, is influenced by changes occurring in wider economic, social, cultural and geopolitical relations. Figure 4 offers an illustration of a revised model that emerged from a series of discussions among the authors while drafting this commentary, and could be used as a starting point for such a consensus process. This model retains the core features of the initial models of Haglund and Svanström and Dahlgren and Whitehead with a hierarchical structuring of determinants, but adds further insights introduced in the Östgöta model (e.g. the inclusion of psychological factors and a depiction of biological mechanisms), as well as what the authors deem as missing or insufficiently emphasised determinants, such as IT and existential aspects. The outer ‘environment perspective’ ring and the arrow at the bottom are directly related to the Sundsvall statement which notes how supportive environments are of paramount importance for health and that “action to create supportive environments has many dimensions: physical, social, spiritual, economic and political. Each of these dimensions is inextricably linked to the others in a dynamic interaction [40].”

## Conclusions

The need to visualise the interactions between various determinants of health continues to drive a demand internationally for rainbow models. Their widespread popularity and utility confirm that they are an important Nordic contribution to wider public health reasoning. Substantial developments have occurred both in our understanding of the determinants of health and public health practices since the original model first began to take shape in Danish sand. We conclude that the time has come for an updated model. As a starting point for this work, we suggest a model that attempts to elucidate and preserve the core value of older rainbow determinants of health models while taking account of newly emerging challenges to health, as well as new knowledge on factors earlier not seen as relevant.
